# Clinical Relevance of Torque Teno Virus (TTV) in HIV/HCV Coinfected and HCV Monoinfected Patients Treated with Direct-Acting Antiviral Therapy

**DOI:** 10.3390/jcm10102092

**Published:** 2021-05-13

**Authors:** Daniele Lapa, Paola Del Porto, Claudia Minosse, Gianpiero D’Offizi, Andrea Antinori, Maria Rosaria Capobianchi, Ubaldo Visco-Comandini, Fiona McPhee, Anna Rosa Garbuglia, Mauro Zaccarelli

**Affiliations:** 1 Laboratory of Virology, “Lazzaro Spallanzani” National Institute for Infectious Diseases, IRCCS, 00149 Rome, Italy; daniele.lapa@inmi.it (D.L.); claudia.minosse@inmi.it (C.M.); maria.capobianchi@inmi.it (M.R.C.); 2 Department of Biology and Biotechnology “C. Darwin”, Sapienza University, 00185 Rome, Italy; paola.delporto@uniroma1.it; 3 Hepatology and Infectious Diseases Unit, “Lazzaro Spallanzani” National Institute for Infectious Diseases IRCCS, 00149 Rome, Italy; gianpiero.doffizi@inmi.it (G.D.); Ubaldo.viscocomandini@inmi.it (U.V.-C.); 4 Clinical Department, “Lazzaro Spallanzani ” National Institute for Infectious Diseases, IRCCS, 00149 Rome, Italy; andrea.antinori@inmi.it (A.A.); mauro.zaccarelli@inmi.it (M.Z.); 5 Bristol-Myers Squibb, Cambridge, MA 02142, USA; fiona.mcphee@bms.com

**Keywords:** Hepatitis C virus, Torque Teno virus (TTV), DAA therapy, cytokines, HIV/HCV coinfection

## Abstract

Torque Teno virus (TTV) is a ubiquitous virus that causes chronic infection in humans with unknown clinical consequences. Here, we investigated the influence of TTV infection on HCV direct-acting antiviral (DAA) efficacy in HIV/HCV coinfected and HCV monoinfected patients as controls. Of 92 study patients, 79.3% were TTV DNA positive; untreated patients exhibited a significantly higher proportion of TTV DNA-positivity vs. sustained virological response (SVR) patients (100.0% vs. 65.2%, *p* < 0.001), while TTV positivity was not significant in DAA failure patients vs. SVR patients despite HIV/HCV coinfection. TTV DNA viral load was higher among HCV monoinfected patients vs. HIV/HCV coinfected, although marginally significant (*p* = 0.074) and no significant viral load difference was detected between DAA failures and SVR patients, while untreated vs. SVR patients had a significantly higher viral load (19,884, IQR 5977–333,534, vs. 469, IQR 10–4124, *p* = 0.004). Alpha-genogroup 3 TTV was the most prevalent genetic group, and no specific strain or genogroup was observed in relapser patients. Among HIV/HCV patients with HCV RNA detectable at end of treatment (EOT), TTV DNA was detected in 9/17 treatment responder patients and 3/5 relapser patients, thus, TTV infection does not appear to influence the control HCV viremia after EOT. Levels of IL-6 IL-4, and CD14 were not significantly different between TTV PCR-positive and -negative patients. These results suggest no association between TTV DNA positivity or viral load and HCV DAA failure whether patients were HIV/HCV coinfected or HCV monoinfected.

## 1. Introduction

Torque Teno virus (TTV) was first identified in a Japanese patient who died from fulminant hepatitis with unknown etiology [1]. TTVs are ubiquitous, genetically heterogeneous, non-enveloped, single-stranded, negative-sense circular DNA viruses with a 3.8 Kb genome. The genome includes 3 coding regions (ORF1, ORF2, ORF3) and a well-conserved untranslated region (UTR) divided into UTRA and UTRB located in 5′ and 3′ GC-rich regions, respectively [2]. TTV belongs to the alphatorquevirus genus, which is part of the *Anelloviridae* family of viruses. Other anellovirus genera that infect humans include the betatorquevirus, Torque Teno Mini virus (TTMV), and the gammatorquevirus, Torque Teno Midi virus (TTDV). To date, 76 species associated with the three noted genera have been identified [3]. Classification into genera is largely based on species-specificity and genome size. TTV belongs to the alphatorquetenovirus genus, which includes 39 genotypes [4,5]. Fourteen genotypes are significant components of the human virome [6]. Phylogenetically, seven genogroups (alpha-genogroups) have been identified [5].

The clinical consequences of TTV infection remain unknown [2,7,8]. TTV infection is chronic in most cases; however, our understanding of the pathogenicity of long-term infections with one or several TTV isolates is minimal. The large circulation within the general population suggests that TTV does not represent a principal etiological agent of a specific disease, but it could be a co-factor favoring the development of a pathologic status. For example, co-infection of human papillomavirus (HPV) with TTV genotype 1 has been associated with poor prognosis in laryngeal cancer, even though it is unknown whether and how TTV can be involved in cancer development [9]. TTV DNA was detected in lymphocytes and lymphomas from patients with B-cell and Hodgkin’s lymphoma [10]. The recent development of viral metagenomic approaches has allowed for better characterization of the genetic diversity of *Anelloviridae*. Indeed, metagenomics facilitated the identification of new *Anelloviridae* genotypes in patients suffering from various diseases, including Kawasaki disease, brain cancer, encephalitis, and periodontitis [11,12,13]. The blood is considered the main route of transmission [14,15,16,17,18].

It has been detected in blood, serum, and breast milk, although this virus shows tropism towards specific cells such as hepatocytes. Okamoto et al. reported that TTV titers were 10–100 fold higher in liver tissue than in serum from patients with transfusion-acquired hepatitis of unknown etiology [19], Asim et al. observed that viral burden in the liver tissue was three times higher than that observed in plasma [20]. Even though TTV has been associated with fulminant non-A, non-G hepatitis, its role in chronic hepatitis is questionable. Reports from several studies suggested no relationship between TTV positivity and liver damage in blood donors [21] or ALT elevation in patients with chronic viral hepatitis [22,23]. TTV is also able to replicate in the bone marrow and mononuclear cells, including T lymphocytes [24,25]. It can interact with innate immune cells by stimulating Toll-like receptor 9 and by inhibiting the NF-kB pathway [26]. TTV viral load is dramatically higher in transplant patients or those with sepsis, although the contribution of the virus to morbidity is unknown. More likely, TTV could associate with other viruses like Epstein-Barr virus (EBV) or Cytomegalovirus (CMV) to induce a pathologic status. In HIV-positive patients, TTV DNA titer has been inversely correlated with CD4 T cell counts [27] and an increase in TTV DNA load was observed in patients who progress to AIDS [28]. 

However, other authors could not establish any correlation between TTV DNA positivity and CD4, CD8 T cell counts or HIV viral load in asymptomatic and symptomatic patients [29,30,31]. In mononuclear infected cells, TTV induces the production of interferon-gamma and tumor necrosis factor-α and increases the concentration of interleukins such as IL-12, IL-28, IL-29, or chemokines (CCL7) [32]. Reduced survival of CD4 T cells has been observed in patients with high TTV DNA viral load [33], although TTV effects on adaptive immunity have not yet been established. Potential effects of TTV infection in HIV/HCV coinfected patients treated with HCV direct acting antivirals therapy (DAA) has not been explored. The introduction of HCV DAA therapies for treating HIV/HCV coinfected patients has produced SVR rates close to 100% [34]. When preexisting drug-resistant substitutions are not a factor, virologic failures to DAA therapy in this group of patients are still a matter of debate. Several factors have been considered as relevant for the success of DAA therapy, including immune reconstitution status, drug user addiction, and the extent of liver damage [35,36,37].

Our study aimed to investigate whether the presence of TTV positivity may influence sustained virological response (SVR) in HIV/HCV coinfected or HCV monoinfected patients treated with DAA therapy. In addition, we evaluated the association between TTV infection and the levels of immune and inflammatory markers in sera from DAA-treated patients analyzed by treatment outcome.

## 2. Materials and Methods

### 2.1. Population and Sample Collection

This retrospective study was performed at the Laboratory of Virology of the National Institute for Infectious Diseases INMI L Spallanzani.

Overall, 92 HCV-infected patients were tested for TTV DNA by PCR and included in the analysis. To reduce selection bias, the following patients were included: (i) HCV patients treated with DAA in our Institute facilities between 2016 and 2019 and, subsequently, failed treatment (relapser); (ii) patients treated with DAA between 2016 and 2018 and achieved an SVR and returned to our Institute outpatient clinic in 2018 for a follow-up visit; and (iii) untreated HCV patients who also attended our Institute outpatient clinic for a medical check-up to assess their clinical status for starting DAA therapy. All patients completed informed consent forms for the study. This study was conducted following Good Clinical practice guidelines and was approved by the Local Research Ethics Committee of INMI L Spallanzani IRCCS Hospital (protocol code 55/08).

#### 2.1.1. HCV RNA Detection

HCV RNA viral load was quantified by a commercial real-time RT-PCR assay (RealTime™ HCV, Abbott Molecular Inc, Des Plaines, IL, USA) as specified by the manufacturer. The lower limit of detection (LLOD) was 12 IU/mL. HCV genotypes were assigned using the Abbott RealTime HCV Genotype II Assay (Abbott Molecular Inc, Des Plaines, IL, USA). Samples with an HCV RNA reading of “detected < 12 IU/mL”, were confirmed with an ultrasensitive method as previously described [38]. HCV RNA levels were measured at baseline, end of treatment (EOT), 4, 12, and 18 weeks after completion of treatment.

Residual plasma samples were collected and stored at −80 °C and used for TTV DNA detection and cytokine level determination.

#### 2.1.2. Real-Time Quantitation of TTV DNA Load

Viral DNA was extracted from 400 μL of plasma samples using the QIASYMPHONY instrument (QIAGEN, Hilden, Germany). Nucleic acids were eluted in 60 μL of AVE elution buffer (QIAGEN, Hilden, Germany).

TTV DNA viral load quantification was achieved by employing the real-time TTV R-GENE assay targeting a highly conserved segment of the untranslated region (UTR) of the viral genome and using the Rotorgene Q instruments (QIAGEN) following the manufacturer’s instructions (BioMérieux, France) [39]. The assay limit of detection is 167 copies/mL and the linearity range is 1.61–10.61 Log copies/mL [39]. This assay can detect and quantify a broad range of human alpha-torquetenovirus genotypes (TTV genotypes 1/3/6–8/10/12/15/16/19/27/28) [39,40] belonging to genogroups 1–5. All DAA-treated patients had their serum tested at EOT.

### 2.2. TTV DNA Amplification and Phylogenetic Analysis

Eluted nucleic acids (10 μL) were used for TTV-DNA detection by semi-nested PCR using primers designed against the untranslated genome B (UTRB) region, as previously described [41]. Primers were designed using the TTV TA278 isolate genome fragment spanning nucleotides 3362–3739 (Genbank accession number AB008394). For first-round PCR, the sense primer NG148 5′-CGAAAGTGAGTGGGGCCAGACTTC-3′ and antisense primer NG065 5′-GCCGACGGTTTTTTGGCGCCTTTTTTC-3′ were used under the following PCR conditions: 95 °C for 15 min, followed by 35 cycles of denaturation at 94 °C for 30 s, annealing at 60 °C for 45 s, extension at 72 °C for 45 s, with a final extension at 72 °C for 45 s. Second-round PCR was performed using the same PCR conditions with an internal sense primer NG149 5′-CCATAAGGCCTTTATCTTGCC-3′ and the antisense primer NG065. TaqGold polymerase (Applied Biosystems, Foster City, CA, USA) was employed for both rounds of amplification. PCR products were analyzed by agarose gel electrophoresis and Sanger sequencing (3500XL Genetic Analyzer, Applied Biosystems, Foster City, CA, USA). Forward and reverse strand electropherogram results were edited and assembled using the BioEdit sequence alignment editor software version 7.1.9 (Available online: http://www.mbio.ncsu.edu/B (accessed on 16 December 2020) [42].DNA sequences were checked against the GenBank nucleotide database and were confirmed to be TTV using the Basic Local Alignment Search Tool (BLAST) from the National Center of Biotechnology Information (NCBI, National Institute of Health, USA). INMI sequences were aligned with UTR sequences of alpha-genogroups 1–5 retrieved from GenBank. Sequence similarity was assessed using CLUSTALW [43]. Phylogenetic analysis was performed using the maximum likelihood method and the MEGA software package v.10 (Available online: http://www.megasoftware.net, accessed on 10 December 2020) [44]. The topological robustness of the tree was evaluated using a bootstrap analysis with 1000 replicates.

**Figure 1:**
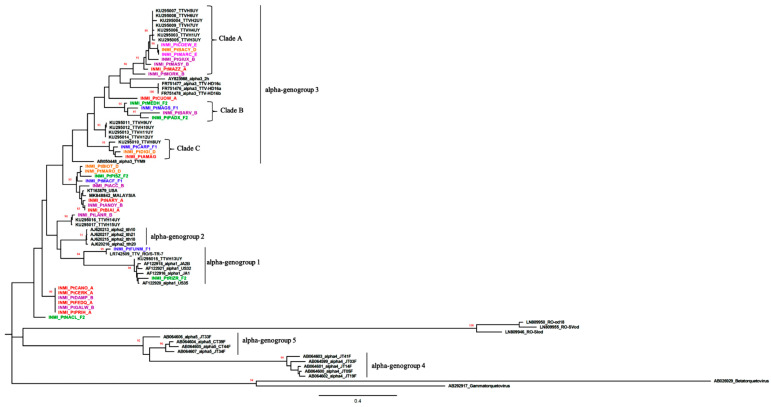
Phylogenetic analysis of TTV UTR B (3′UTR) region from patient-derived samples. Thirty-three patient-derived nucleotide sequences and representative TTV genogroup sequences, chosen following the original publications [8,14,41,45,46] and ICVT [47] were used for the construction of a Maximum-likelihood method tree that was visualized with Fig Tree (Available online: http://tree.bio.ed.ac.uk/software/figtree/ (accessed on 22 December 2020)). Bootstrap values > 80% are indicated on respective branches. Scale bar represents nucleotide substitutions per site. TTV Italian strains were indicated as INMI. Patients included in group A are in red, in group B in violet, group D in orange, group E in pink, group F1 in blue, and group F2 in green. Reference sequences are indicated by their GenBank accession number, genogroup, and name of the isolate if available.The list of all GenBank accession numbers is reported in Supplemental Materials Tables S1 and S2.

Genogroups 6 and 7 were not included in the phylogenetic analysis because the UTR of these genogroups was not available in GenBank (see Figure 1). AB290917, a sequence belonging to gammatorquevirus, was considered as the representative for this group. Genogroups for the INMI sequences were estimated from the Fig tree based on the clustering with reference TTV sequences.

All samples (n = 23) identified as being TTV DNA negative by the Biomérieux kit were also shown to be TTV DNA negative when retested using our in-house developed UTR B PCR assay.

### 2.3. Quantification of Cytokines in Sera

Patient-derived sera were analyzed using enzyme-linked immunosorbent assay (ELISA) kits for IFN-γ, CD14, IL4, and IL6 (R&D Systems, Inc, Minneapolis, MN, USA), respectively, according to the manufacturer’s instructions. Briefly, diluted serum samples and standards were added to plate wells in duplicate and incubated at room temperature. Biotin-conjugated detection antibody was added at room temperature. Biotin-conjugated detection antibody was added followed by avidin-conjugated horseradish-peroxidase. The incubation time varied for each cytokine (see manufacturer’s instructions). Plates were developed using tetramethylbenzidine (TMB) substrate and the reaction was quenched by adding a stop solution. Colorimetric changes of the enzyme substrate were detected at 450 nm wavelength using a microplate reader. Assay limits detection were: 8 pg/mL for INF-γ, 125 pg/mL for CD14, 0.11 pg/mL for IL4, and 0.031 pg/mL for IL6.

### 2.4. Statistical Analysis

Statistical analysis was performed using SPSS (v. 25.0) statistical software (IBM Corp. Released 2017. IBM SPSS Statistics for Windows, Version 25.0. Armonk, NY, USA). The association with TTV PCR detection and TTV viral load (copies/mL) with patient demographics, clinical and laboratory variables related to HIV and HCV infection were assessed using the Fisher exact test and ANOVA test, respectively. Mean and standard deviation (SD) were used to compare patients with continuous variables. A *p*-value < 0.05 was considered statistically significant. Continuous variables (age and CD4 T cell count) were categorized to evaluate any association with TTV PCR positivity and viral load (TTV PCR test) and included in the database.

Age was selected at the cut-off that showed the highest difference with TTV positivity (55 years); for CD4 T cell count, the standard cut-offs used to monitor HIV infection were evaluated (e.g., 200, 350, and 500). Since the CD4 T cell count was generally high in these patients, the 500 count cut-off was also included as a variable parameter.

Patients were classified in subgroups according to HIV sero-status, HCV DAA treatment status, and treatment outcome at EOT and post-therapy.

Group A included HIV/HCV patients treated with HCV DAA and had HCV RNA <12 IU/mL at EOT; 5/22 (27.3%) relapsed after treatment. Group B included HIV/HCV coinfected patients treated with DAA, and had undetectable HCV RNA at EOT, and achieved SVR. Group C included HIV/HCV coinfected patients treated with DAA, who had undetectable HCV RNA at EOT and then relapsed. Group D included HCV monoinfected patients treated with DAA, and had undetectable HCV RNA at EOT, and achieved SVR. Group E included HCV monoinfected patients, who had undetectable HCV RNA at EOT before relapsing. A control group of 24 untreated HCV patients was split between those who were HIV/HCV coinfected (Group F1) and those who were HCV monoinfected (Group F2).

Finally, the association between cytokine values and TTV PCR positivity was also assessed comparing mean values (SD) and statistical significance between mean distribution with the ANOVA test, however, if non-homogeneity of variance was assumed (using Levene’s test for homogeneity of variance), a nonparametric test was used.

## 3. Results

### 3.1. Virological and Immunological Results

The clinical characteristics of patients included in the study, analyzed overall and by both HCV monoinfected and HIV/HCV coinfected status, are described in Table 1. As shown, most (88.0%) were males with a median age of 44 years and over two-thirds were HIV/HCV coinfected patients currently receiving antiretroviral therapy. Regarding HCV status, 46 patients (50%) achieved SVR, 22 (23.9%) failed DAA therapy, and 24 (26.1%) never received HCV treatment. HCV genotype (GT)1a was the most predominant genotype (45.7%), and 30.4% of all patients had cirrhosis. Whether a patient was monoinfected with HCV or coinfected with HIV/HCV, the distribution of HCV GT and cirrhosis was similar.

TTV DNA PCR positivity was detected in 73 patients (79.3%), with a median TTV DNA viral load of 2362 copies/mL (IQ range 32,829). Among HIV-positive patients, the mean CD4 T cell count was 611, indicative of an acceptable immune status. Among DAA-treated patients, those with cirrhosis were more likely to fail treatment (44.0% vs. 25.6% in non-cirrhotic patients, *p* = 0.098), as well as those with HCV GT3 (54.5% vs. 29.1% in non-cirrhotic patients, GT3 *p* = 0.101).

The relationship between TTV DNA PCR positivity and TTV viral load and other variables of interest are reported in Table 2. The continuous variables (age, CD4 T cell count) were categorized at the best cut-point for association with TTV DNA PCR. The percentage of TTV DNA PCR positivity was significantly lower in HIV/HCV coinfected patients vs. HCV monoinfected patients. Patients with detectable HCV RNA (HCV untreated or DAA failure) showed a higher positivity to TTV DNA than DAA-treated patients achieving SVR. In particular, TTV DNA positivity was significantly higher in untreated vs. SVR patients (100.0% vs. 65.2%, *p* < 0.001), while numerically higher in DAA failures vs. SVR patients (86.4% vs. 65.2%, *p* = 0.06). No significant association was observed between TTV DNA PCR positivity and gender, cirrhosis, HCV-genotype, age, and CD4 T cell count. The TTV DNA viral load was significantly higher in HCV untreated patients vs. SVR patients; however, no significant difference was detected between DAA failures and SVR patients. Finally, TTV DNA viral load was numerically higher among HCV monoinfected patients vs. HIV/HCV coinfected, although not significant (*p* = 0.074). The association of TTV PCR positivity and copies/mL with subgroups A-F1/F2 is reported in Table 3. As shown, HCV monoinfected DAA relapsers (Group E, n = 11), untreated HCV monoinfected (Group F2, n = 11), and HIV/HCV coinfected (Group F1, n = 13) patients were all TTV DNA PCR positive. No significant differences were found when comparing HIV/HCV coinfected subgroups (A, n = 22; B, n = 22; C, n = 6). Moreover, no overall significant differences were observed when comparing TTV DNA PCR levels within subgroups, although HCV monoinfected always exhibited higher TTV PCR levels vs. HIV/HCV coinfected patients irrespective of whether patients: (i) achieved an SVR, (ii) failed DAA therapy, or (iii) were DAA treatment-naive.

We next evaluated for TTV DNA PCR positivity association with serum inflammatory (CD14 and IL-6) and immune markers (IFN-g and IL-4). Levels of the monocyte/macrophage activation marker, CD14, and the pro-inflammatory cytokine, IL-6, were not significantly different between TTV DNA PCR positive and negative patients. Similarly, no difference in mean IL-4 values was observed between the two groups of patients (Table 4). The levels of IFN-ɣ were below the assay detection limit in all analyzed patients.

For completeness, the levels of serum IL-6, IL-4, and CD14 were analyzed by the patient subgroup. For Group C (HIV/HCV DAA relapsers), only IL-6 was evaluated due to limitations in available serum. As reported in Table 5, the levels of IL-6 and CD14 were not significantly different among the patient groups analyzed. Statistically significant associations among groups were found with IL-4 distribution, which ranged between the lowest values for Group B (*p* = 0.011 vs. other groups) to the highest values for Group A (*p* < 0.014 vs. other groups).

Finally, we verified whether TTV DNA positivity could somehow influence the viral rebound in patients with HCV RNA <12 IU/mL at end-of-treatment (EOT; Group A). For the five relapser patients included in Group A, three were TTV DNA positive. In addition, TTV DNA was also present in 9/17 patients who achieved SVR, suggesting that TTV infection is not relevant in the control of HCV viremia after EOT in HIV/HCV coinfected patients.

### 3.2. Phylogenetic Analysis

The UTRB region was successfully amplified in 43 of 73 TTV DNA positive samples detected by the Biomérieux assay. Multiple TTV isolates were detected in 10 samples, probably due to co-infections, complicated sequence identification, thus, phylogenetic analysis was performed using 33 sequences. References for TTV alpha-genogroup 1 (genogroup1) to 5 were included in the phylogenetic analysis (Supplemental Materials Tables S1 and S2). A phylogenetic tree was constructed employing the maximum-likelihood method (Figure 1). TTV alpha-genogroups included genogroup3 (n = 13), genogroup1 (n = 2), and undetermined genogroup strains (n = 18). Genogroup1 strains were observed only in untreated patients: one in the F1 group and the other in the F2 group, while genogroup3 isolates were found in all groups. The genogroup3 strains were grouped into three different clades. Both strains from coinfected patients and those from monoinfected patients were found in all three clades. In clade A, sequences shared a similarity of 81.1%, in clade B, 79.6%, and in clade C, 90.8%. PtINMI_CUOW had a 75.3% similarity with AY823988, an alpha-genogroup3 reference sequence.

Six undetermined strains clustered in the same clade. They showed a similarity of 81.5% with the genotype2 reference sequence AJ20213. INMI_PtNACL had a similarity of 85% with the other six strains and 83.2% identity with AJ62013. INMI_PtRIZR belonged to genogroup1 with a similarity of 95.3% with a USA reference strain, AF122920_Alpha1. The similarity of INMI_PtFUNM was 98.4% with LR742509 from Romania and 71.8% with AF122920_Alpha1. High similarity was observed with isolates from different geographical locations. In particular, INMI_PtAN131 and INMI_PtBIA exhibited 99.1% similarity with MK848842, a strain isolated from a patient with hepatitis in Malaysia, and 98.7% with KT163879, a strain isolated from an HIV patient from the USA.

Moreover, phylogenetic analysis (Figure 1) demonstrated that the distribution of alpha-genogroups was not associated with HIV status.

## 4. Discussion

In this study, we analyzed the impact of TTV infection on SVR achievement both in HIV/HCV coinfected and HCV monoinfected patients. Interestingly, TTV prevalence was lower in HIV/HCV coinfected patients in comparison to HCV monoinfected patients (63.0% vs. 93.1%, *p* = 0.029). This data may be explained by the fact that antiretroviral therapy has been associated with inhibition of TTV replication [31]. Indeed, Devalle et al. observed that after initiating HAART, TTV positivity and genogroup variability were less in HIV-positive patients. This finding is supported by the lower TTV viral load in HIV/HCV coinfected patients (44,004 ± 127,039 copies/mL) vs. HCV monoinfected patients (572,941 ± 2,328,908 copies/mL; *p* = 0.074). The high number of CD4 T cells may also explain the modest TTV viral load detected in HIV/HCV patients. It has been noted in several studies that restoration of the immune response may cause a reduction in TTV viral load/positivity detected in patient sera [27,48,49]. Overall, TTV infection was detected in 79.3% of patients, in agreement with previously reported data [14,41,50,51,52], and no correlation was observed between TTV positivity and achievement of SVR since TTV DNA prevalence was similar between HIV/HCV coinfected groups B and C and HCV monoinfected groups D and E (Table 3). Analysis of gender relationships with TTV was non-conclusive since the majority of patients were males (88%).

No significant differences between the infecting HCV genotype and TTV-PCR positivity or TTV viral load were observed. This suggests TTV infection was not associated with specific HCV genotypes.

Assessment of liver pathology indicated no significant associations between TTV DNA and cirrhosis or a specific HCV genotype. This agrees with Garcia-Alvarez [51] who found that there were no significant differences in TTV viral load between HIV/HCV coinfected patients with various stages of fibrosis.

The majority of patients in our study were infected with TTV genogroup 3 irrespective of HIV serostatus, consistent with previous reports [53,54].

All TTV strains with an undetermined genogroup exhibited high similarity with other TTV strains isolated from different geographical locations that included Malaysia and the USA, suggesting a ubiquitous distribution of these TTV strains (Figure 1). This underscores TTV heterogeneity and suggests that only full-genome sequencing will help assess whether indeterminate isolates are variants or new genotypes.

No patient in our study was infected with TTV genogroup 4. This agrees with Reference [52], where genogroup 4 was not detected in HBV and HCV positive patients.

Presently, the role of TTV infection in regulating host innate and adaptive immune responses is unclear. In vitro studies have demonstrated that different molecules from TTV can positively or negatively regulate the expression of key inflammatory mediators at the cellular level. Indeed, Rocchi et al. demonstrated that DNA representing TTV genogroup 4 TTV (ViPiSAL) activated the production of IL-6 and IFN-ү through the stimulation of TLR9 in murine spleen cells [55], while Zheng et al. reported that TTV ORF2 protein suppressed the activity of NF-kB by inhibiting its translocation to the cell nucleus and subsequent transcription of genes, such as IL-6, IL-8, and cyclo-oxygenase-2 [26].

The observed lack of a significant association between TTV DNA positivity and markers of inflammation (IL-6, CD14) or adaptive immune responses (IFN-ɣ, IL-4) in untreated or HCV DAA-treated HIV/HCV coinfected and HCV monoinfected patients do not support a role for TTV in affecting innate or adaptive immune responses in vivo. However, it cannot be ruled out that the similar quantities of cytokines detected in sera from TTV-negative and -positive patients may be a consequence of the low immunostimulatory properties of infecting viral strains. In particular, none of our patients were infected with TTV genogroup 4, which, according to the CpG index, has been predicted to be the most immunostimulatory TTV genogroup among genogroups 1 through 5 [55].

Overall, our results demonstrated that HIV-positive patients had a TTV DNA viral load similar to those described in healthy donors [39,56], indicating both an immune reconstitution and effectiveness of antiviral therapy in inhibition of TTV replication. In our study, no TTV DNA viral load cut-off correlated with HCV DAA treatment failure, unlike the findings observed in transplanted patients, where a TTV viral load cut-off of 9.3 Log copies/mL was predictive for the development of infection [57,58], and a positive correlation was observed between TTV DNA viral load and doses of prednisolone and mycophenolate mofetil.

There were a few limitations in the present study. The first was attributed to the single center study design and the power of subgroup analyses associated with a small sample size. Second, TTV DNA viral load evaluations were performed only on EOT samples from HCV DAA-treated patients since samples at additional visits were not available. Third, the heterogeneity in our tested patient population with respect to the infecting HCV genotype and DAA treatment may have introduced confounding variables. However, bias related to DAA treatment outcome was limited since all patients visiting our center during a defined period were asked about participating in our study.

## 5. Conclusions

In conclusion, an association between TTV viral load and HCV DAA failure was not observed in HIV/HCV coinfected and HCV monoinfected patients, respectively. Additionally, TTV viral load and HCV DAA treatment success were independent of the infecting TTV genogroup harbored by patients.

## Figures and Tables

**Table 1:** General characteristics of the study population.

	All Patients n (%)	HCV Mono-Infected n (%)	HIV/HCV Coinfected n (%)
Overall	92 (100.0%)	29 (31.5%)	63 (68.5%)
Gender			
Male	81 (88.0%)	21 (72.4%)	60 (95.2%)
Female	11 (19%)	8 (27.6%)	3 (4.8%)
TTV DNA-PCR positive	73 (79.3%)	27 (93.1%)	46 (73%)
HCV treatment response			
SVR	46 (50.0%)	7 (24.1%)	39 (61.9%)
Untreated	24 (26.1%)	11 (37.9%)	13 (20.6%)
DAA failure	22 (23.9%)	11 (37.9%)	11 (17.5%)
Cirrhosis	28 (30.4%)	10 (34.5%)	18 (28.6%)
HCV genotype			
1a	42 (45.7%)	9 (31.0%)	33 (52.4%)
1b	15 (16.3%)	7 (24.1%)	8 (12.7%)
2	9 (5.4%)	3 (10.3%)	2 (3.2%)
3	13 (14.1%)	6 (20.7%)	7 (11.1%)
4	3 (3.3%)	3 (10.3%)	11 (17.5%)
Other/unknown	3 (3.3%)	1 (3.4%)	2 (3.2%)
Age (median, IQ-range)	53 (49–56)	53 (48–59)	53 (49–56)
TTV DNA, copies/mL (median, IQ range)	2259 (50–31,446)	29,715 (6578–55,841)	778 (13–5777)
CD4 T cell count * (median, IQ range)			611 (359–950)

* CD4 T cell count in 59 HIV/HCV positive patients; n, number.

**Table 2:** Univariable association of TTV DNA positivity and copies/mL with viral markers.

	TTV DNA PCR Pos (%)	*p*-Value *	TTV DNA PCR cp/mL, Median (IQ Range)	*p*-Value *
Gender				
Male	63/81 (77.8%)		1351 (39–31,071)	
Female	10/11 (90.9%)	0.449	31,446 (10,067–396,782)	0.894
HIV				
HIV/HCV coinfected	46/63 (63.0%)		881 (15–5818)	
HCV monoinfected	27/29 (93.1%)	0.029	31,446 (6857–114,056)	0.232 **
HCV status				
SVR	30 /46 (65.2%)		469 (10–4124)	
Untreated	24/24 (100.0%)	<0.001 ***	19,884 (5977–333,534)	0.04 **** (**)
DAA failure	19/22 (86.4%)	0.06 ***	4915 (450–64,535)	
Cirrhosis				
Yes	23/28 (82.1%)		6934 (558–80,913)	
No	50/64 (78.1%)	0.784	1419 (42–28,914)	0.523
HCV genotype				
1a	34/42 (45.7%)		4227 (155–30,609)	
1b	13/15 (86.7%)		8079 (575–55,841)	
2	3/5 (60.0%)		50 (10–264,129)	
3	11/13 (84.6%)		2259 (32–249,452)	
4	9/14 (64.3%)		248 (10–17,882)	
Other/unknown	3/3 (100.0%)	0.727	10,067 (1079–10,067)	0.366 (1a vs. oth) **
Age				
≥55	29/33 (87.9%)		6935 (566–28,114)	
<55	44/59 (60.3%)	0.181	1514 (18–36,679)	0.216 **
CD4 T cell count				
<500	16/19 (84.2%)		469 (10–5337)	
≥500	26/40 (65.0%)	0.218	1232 (159–11,399)	0.311

cp, copies; pos, positive; oth, other; * *p*-value calculated using the Fisher exact test or the ANOVA test, ** if non-homogeneity of variance was assumed by the Levene’s test, a nonparametric test was used, *** *p* < 0.001 for untreated vs. SVR patients, *p* = 0.06 for DAA failure vs. SVR patients, **** *p* = 0.04 for HCV untreated vs. responders.

**Table 3:** Association of TTV DNA positivity and copies/mL with subgroups of patients.

	TTV DNA PCR Positive n. /Total (%)	TTV DNA PCR Copies/mLMedian (IQ Range)
Group A	12/22 (54.5%)	95 (10–2204)
Group B	16/22 (72.7%)	830 (126–2590)
Group C	5/6 (83.3%)	348 (33–4677)
Group D	5/7 (71.4%)	10,067 (10–29,715)
Group E	11/11 (100.0%)	52,781 (8050–137,494)
Group F1	13/13 (100.0%)	12,999 (2581–305,659)
Group F2	11/11 (100.0%)	31,446 (7136–396,782)

Cp, copies; *p* = 0.002 at chi-square for trend. Group A, HIV/HCV coinfected patients with detectable HCV RNA at end of treatment (EOT); Group B, HIV/HCV coinfected patients who achieved a sustained virological response (SVR); Group C, HIV/HCV coinfected DAA relapsers; Group D, HCV monoinfected patients who achieved SVR; Group E, HCV monoinfected DAA relapsers; Group F1, untreated HIV/HCV coinfected patients; Group F2, untreated HCV monoinfected patients.

**Table 4:** Association of cytokines with TTV DNA positivity.

	TTV DNA PCR Pos Median (IQ Range)	TTV DNA PCR Neg Median (IQ Range)	*p*-Value *
IL-6	2.87 (1.70–4.56)	2.07 (1.82–5.77)	0.810
IL-4	0.00 (0.00–0.29)	0.56 (0.00–1.96)	0.200
CD14	8577 (6822–10,273)	9589 (7573–10,842)	0.615

pos, positive; neg, negative; * *p*-value calculated using the ANOVA test since equal variance was assumed by Levene’s test for equality of variance.

**Table 5:** Association of cytokines with patient subgroups.

	IL-6 Median (IQ Range)	IL-4 * Median (IQ Range)	CD14 Median (IQ Range)
Group A	2.01 (1.37–5.46)	1.24 (0.00–2.33) **	9009 (734–11,450)
Group B	3.18 (2.58–4.64)	0.00 (0.00–0.13) ***	9326 (7814–10,928)
Group C	1.91 (0.00–3.38)	ND	ND
Group D	4.60 (1.47–10.13)	0.28 (0.03–1.13)	8625 (7405–10,782)
Group E	2.96 (1.31–6.28)	0.00 (0.00–0.40)	8724 (6344–10,778)
Group F1	2.71 (1.62–3.67)	0.00 (0.00–0.87)	8717 (8132–13,177)
Group F2	3.68 (2.25–7.60)	0.18 (0.02–0.59)	8577 (8300–12,459)

ND, not determined due to insufficient sample; * *p*-value was calculated only on IL-4 results; ** *p* < 0.014 for group A vs. other groups; *** *p* = 0.011 for group B vs. other groups; Group A, HIV/HCV coinfected patients with detectable HCV RNA at end of treatment (EOT); Group B, HIV/HCV coinfected patients achieving sustained virological response (SVR); Group C, HIV/HCV coinfected DAA relapsers; Group D, HCV monoinfected patients achieving SVR; Group E, HCV monoinfected DAA relapsers; Group F1, untreated HIV/HCV coinfected patients; Group F2, untreated HCV monoinfected patients.

## Data Availability

The data underlying this article will be shared upon request to the corresponding author.

## Supplementary Materials

The following are available online at https://www.mdpi.com/article/10.3390/jcm10102092/s1, Table S1: TTV strains included in the phylogenetic analysis, Table S2: GenBank accession numbers of patients sequences described in Phylogenetic tree.
