# Supplementary material for: Clinical Relevance of Torque Teno Virus (TTV) in HIV/HCV Coinfected and HCV Monoinfected Patients Treated with Direct-Acting Antiviral Therapy
Source: J Clin Med. 2021 May 13;10(10):2092. doi: 10.3390/jcm10102092 (PMC8152458; doi:10.3390/jcm10102092)
Supplement: Supplementary file 1 [file jcm-10-02092-s001.zip › jcm-1212970-SI.pdf]

Table S1: TTV strains included in phylogenetic analysis

acc. number	Isolate	genogroup	country
AF122916	JA1	Alpha1	USA
AF122918	JA2B	Alpha1	USA
AF122920	US35	Alpha1	USA
AF122921	US32	Alpha1	USA
AJ620213	tth10	Alpha2	Germany
AJ620215	tth18	Alpha2	Germany
AJ620216	tth20	Alpha2	Germany
AJ620217	tth21	Alpha2	Germany
AY823988	2h	Alpha3	Brazil
FR751476	TTV-HD16a	Alpha3	Germany
FR751477	TTV-HD16c	Alpha3	Germany
FR751478	TTV-HD16b	Alpha3	Germany
AB050448	TYM9	Alpha3	Japan
AB064599	JT03F	Alpha4	Japan
AB064600	JT05F	Alpha4	Japan
AB064601	JT14F	Alpha4	Japan
AB064602	JT19F	Alpha4	Japan
AB064603	JT41F	Alpha4	Japan
AB064604	CT39F	Alpha5	Japan
AB064605	CT44F	Alpha5	Japan
AB064606	JT33F	Alpha5	Japan
AB064607	JT34F	Alpha5	Japan
AB026929	TLMV-CBD203	Betatorquetovirus	n.d
AB290917	MD1-032	Gammatorquetovirus	Japan
KU295003	TTVH1UY	n.d	Uruguay
KU295004	TTVH2UY	n.d	Uruguay
KU295005	TTVH3UY	n.d	Uruguay
KU295006	TTVH4UY	n.d	Uruguay
KU295007	TTVH5UY	n.d	Uruguay
KU295008	TTVH6UY	n.d	Uruguay
KU295009	TTVH7UY	n.d	Uruguay
KU295010	TTVH8UY	n.d	Uruguay
KU295011	TTVH9UY	n.d	Uruguay
KU295012	TTVH10UY	n.d	Uruguay
KU295013	TTVH11UY	n.d	Uruguay
KU295014	TTVH12UY	n.d	Uruguay
KU295015	TTVH13UY	n.d	Uruguay
KU295016	TTVH14UY	n.d	Uruguay
KU295017	TTVH15UY	n.d	Uruguay
LN809946	RO-SIod	n.d	Romania
LN809950	RO-od18	n.d	Romania
LN809955	RO-SVod	n.d	Romania
KT163879	P3	n.d	USA
MK848842	TTVMY HC19	n.d	Malaysia
LR742509	RO/S-TR-7	n.d	Romania

n.d., genogroup not reported

Table S2. GenBank accession numbers of patients sequences described in Phylogenetic tree

GenBank acc. number	Patient code	country
MW495822	INMI_PtACC	Italy
MW524862	INMI_PtMASY	Italy
MW524863	INMI_PtCARP	Italy
MW535245	INMI_PtFUNM	Italy
MW535246	INMI_PtMACF	Italy
MW535247	INMI_PtMAGS	Italy
MW535248	INMI_PtMEDH	Italy
MW535249	INMI_PtNACL	Italy
MW535250	INMI_PtPADX	Italy
MW535251	INMI_PtPISZ	Italy
MW535252	INMI_PtRIZR	Italy
MW535253	INMI_PtAMAG	Italy
MW535254	INMI_PtBACY	Italy
MW535255	INMI_PtBARV	Italy
MW535256	INMI_PtBIOT	Italy
MW550303	INMI_PtCOEW	Italy
MW550304	INMI_PtDIGI	Italy
MW550305	INMI_PtGIUX	Italy
MW550306	INMI_PtMARO	Italy
MW570506	INMI_PtMARC	Italy
MW570507	INMI_PtANOY	Italy
MW570508	INMI_PtBIAI	Italy
MW570509	INMI_PtCANO	Italy
MW570510	INMI_PtCERK	Italy
MW570511	INMI_PtCUOW	Italy
MW570512	INMI_PtDAMP	Italy
MW570513	INMI_PtFEDQ	Italy
MW570514	INMI_PtGALW	Italy
MW570515	INMI_PtLANR	Italy
MW570516	INMI_PtMAZZ	Italy
MW570517	INMI_PtMORK	Italy
MW570518	INMI_PtNARY	Italy
MW570519	INMI_PtPRIH	Italy
